# Impact of a Switch to Plant-Based Foods That Visually and Functionally Mimic Animal-Source Meat and Dairy Milk for the Australian Population—A Dietary Modelling Study

**DOI:** 10.3390/nu15081825

**Published:** 2023-04-10

**Authors:** Anita S. Lawrence, Huiying Huang, Brittany J. Johnson, Thomas P. Wycherley

**Affiliations:** 1 School of Agriculture and Food, University of Melbourne, Parkville, VIC 3010, Australia; 2 Caring Futures Institute, College of Nursing and Health Sciences, Flinders University, Bedford Park, SA 5042, Australia; 3 Alliance for Research in Exercise, Nutrition and Activity (ARENA), University of South Australia, Adelaide, SA 5001, Australia

**Keywords:** dietary simulation, dietary transition, meat alternatives, meat analogues, meat substitutes, milk alternatives, milk analogues, nutritional inadequacy, sustainable

## Abstract

Sales of plant-based ‘meat’ and ‘milk’—products that mimic the visual and functional characteristics of animal-source foods—have increased rapidly during the past decade and are predicted to continue to increase. As plant-based ‘meat’ and ‘milk’ are nutritionally dissimilar to the animal-source originals, this study aimed to estimate the nutritional implications for the Australian population of substituting ‘Easily Swappable’ animal-source meat and dairy milk with plant-based imitation products. Computer simulation modelling was undertaken using dietary intake data collected in 2011–12 from a nationally representative survey sample. Conservative and Accelerated dietary transition scenarios were modelled in which various amounts of dairy milk and animal-source meat were replaced with plant-based ‘milk’ and plant-based ‘meat’, for the entire population and for various sub-populations. The scenarios were based on sales reports and economic projections. Modelling revealed that the intake of nutrients already at risk of inadequate intake, such as iodine and vitamin B12 (particularly for females), zinc (particularly for males) and n-3 long-chain fatty acids (for adults), would likely be adversely impacted in an Accelerated scenario. In conclusion, widespread replacement of dairy milk and animal-source meat with plant-based ‘milk’ and ‘meat’ may increase the risk of nutritional inadequacies in the Australian population. Messages and policy actions promoting the transition to more environmentally sustainable diets should be designed to avoid such adverse nutritional impacts.

## 1. Introduction

With predicted global population growth and the urgent need to mitigate climate change, the transition to dietary patterns that are both healthy and environmentally sustainable is a key challenge of our time. Leading authoritative organizations such as the Intergovernmental Panel on Climate Change (IPCC) have concluded that reducing the intake of animal-source food such as meat and dairy and increased consumption of plant-based foods could help to mitigate greenhouse gas emissions [1]. Policy instruments highlighted by the IPCC to encourage populations to adopt more plant-based and less animal-based diets include taxes on food products, food regulations and labelling, procurement policies, regulations about the marketing of food, the development of sustainable dietary guidelines and ‘green nudging’ [1]. Already, a dietary transition is evident—for example, the ‘Meatless Monday’ and ‘Meat-free Monday’ campaigns are currently operating in over 40 countries [2]. Using ‘big data’ to determine the environmental impact of ingredients used in food manufacturing, a UK study estimated that plant-based sausages had an environmental impact approximately 3–10 times lower than animal-source-meat-containing sausages [3].

While diets high in ‘coarse’ grains, pulses, fruit, vegetables, nuts and seeds and low in animal-source and energy-dense, nutrient-poor foods are recommended to reduce greenhouse gas emissions and improve planetary health [1,4], it is sales of plant-based products mimicking the functionality and appearance of animal-source foods that have exhibited the most pronounced increase in recent years in many western countries, including Australia [5,6,7]. There was a five-fold increase in the number of plant-based ‘meat’ products available in Australian supermarkets between 2015 and 2019 [7] and a doubling in demand for plant-based ‘milk’ between 2011 and 2021 [5]. Grocery sales of plant-based ‘meat’ in Australia grew by 46% in 2020, and the range of products available in supermarkets doubled [8]. The increased apparent consumption of plant-based ‘milk’ in Australia between 2018–2019 and 2020–2021 matches the decline in dairy milk consumption (4.0 g/person/d), suggesting a direct substitution of products [9]. Similarly, purchases of ‘meat substitutes’ (which includes vegetarian sausages) increased and those of animal-source meat sausages decreased over these years (+0.3 g vs. −1.0 g/person/d) [9,10].

Although plant-based products mimicking the appearance of meat are easy ‘functional’ replacements for animal-source meat, they are nutritionally dissimilar [10]. Plant-based ‘meat’ products sold in Australia, such as burgers, sausages and mince, are generally lower in energy, fat and saturated fat and fibre than the corresponding animal-source meat products [7,11]. The content of other nutrients, particularly most micronutrients other than sodium, was not determined as the analyses were restricted to nutrients listed on the food packaging. Addressing this evidence gap, a US study compared 37 plant-based ground-beef-like products (mostly burgers) with animal-source meat [12]. That analysis concluded that plant-based ground-beef-like products were ‘good’ or ‘high’ sources of dietary fibre, iron, manganese, copper, folate and niacin while being low in saturated fat but were high in sodium and generally contained less protein, zinc and vitamin B12 than animal-source beef. Evidence from randomised controlled trials assessing the impact of replacing animal-source foods with plant-based versions is limited due to the number of studies, duration of the dietary intervention, number of participants and changes in intake of some nutrients not being comprehensively examined [13,14].

Numerous studies have shown plant-based ‘milks’ to be nutritionally dissimilar, both to each other and to dairy milk [15,16,17,18,19,20,21]. This is partly due to differences in the nutritional content of the base ingredient (e.g., almonds, oats, rice, soy, coconut) as well as differences in the fortification and sweetening practices (which vary between products and between countries) [16]. As with plant-based ‘meats’, the nutrient levels reported in the scientific literature tend to be derived from the food packaging, and accurate information about the content of other nutrients, in particular micronutrients, is limited.

The nutritional implications of switching from animal-source meat and dairy milk to products that, in appearance and functionality, mimic these foods are unlikely to be the same for all population groups. For example, Millennials (people born 1980–2000) have been shown to be more attracted to plant-based alternatives than other age groups [8,22], such as older adults [8,22,23]. In addition, a higher proportion of women than men reported avoiding dairy milk in an Australian survey [24]. According to the most recent Australian national nutrition survey, the proportion of nutritional intake supplied by dairy milk and animal-source meat also varied between population groups, with dairy milk supplying 17.8% of protein intake for 2- to 3-year-olds and 7.4% for adults 71 years and older, but only 5.3% and 5.0% for women and men aged 19–30 years, respectively [25]. 

Dietary modelling is one of several research tools available to assist with the population-wide transition to more environmentally sustainable diets, while maintaining nutritional adequacy. It is particularly useful for predicting future nutritional changes relatively quickly and inexpensively. Once identified, there is the potential to avert adverse changes, for example, by tailoring policies and public health messages. Several dietary modelling studies have assessed the impact of various amounts and types of animal-source meat and/or dairy milk being replaced with various amounts and types of plant-based alternatives in a range of countries and population groups [26,27,28,29]. However, this has often been restricted to an analysis of nutrients listed on food labels, and the differences in intake of some micronutrients have received little attention. There has been no estimation focused on the nutritional implications of the current dietary trend of plant-based foods that visually and functionally ‘mimic’ animal-source meat and dairy milk replacing these animal-source products in the Australian diet. 

The primary aim of the present study was to estimate the nutritional implications for the Australian population of substituting ‘like-for-like’ animal-source meat and dairy milk with plant-based ‘meat’ and plant-based ‘milk’. A secondary aim was to explore the predicted nutritional implications of these substitutions for sub-population groups, namely, young children (2–3 years), young men (19–30 years), young women (19–30 years) and older adults (71 years and over).

## 2. Materials and Methods

### 2.1. Data Source and Preparation

This study was of a microsimulation dietary modelling design, following methods used previously by BJJ and TW, and is reported in line with the ‘Critical appraisal criteria for methodology and reporting quality assessment dietary simulation modelling’ [30]. The study used microdata from the 2011–12 National Nutrition and Physical Activity Survey (NNPAS), part of the 2011–12 Australian Health Survey undertaken between May 2011 and June 2012 [31]. No additional ethics approval was required. In the NNPAS, dietary intake was assessed using the 24 h dietary recall method, and other data were collected from a randomly selected, nationally representative sample (*n* = 12,153) of the Australian population aged 2–90 years of age. For the total population and for each subgroup, de-identified participant dietary record data from the first 24 h recall were weighted against population benchmarks for age, gender and area of usual residence. Weighted dietary intake data were then aggregated for each 8-digit food code and divided by the total population/subgroup-population to derive the per capita average intake quantity of each food item. ‘Base case’ population average nutrient intakes for each food item were derived by linking the food codes (and average intake quantity) to their corresponding national food composition database nutrient profile (AUSNUT 2011–13, containing 5740 foods and beverages [32]). ‘Base cases’ were derived for the total population (age 2+ years) and sub-populations: young children (2–3 years), young women (19–30 years), young men (19–30 years) and older adults (71 years and over). Internal validity was verified through comparison with published data [25].

### 2.2. Identification of ‘Easily Swappable’ Dairy Milk and Animal-Source Meat and ‘Key Nutrients’

The dairy milk and animal-source meat that could be relatively ‘easily swapped’ for like-for-like plant-based ‘milk’ and plant-based ‘meat’ were identified from 8-digit food codes. ‘Easily Swappable Animal-Source Meat’ was defined as beef steak, fillet and mince (codes 18101001–18101273), various cuts of chicken—mainly breast, drumsticks, wings, thighs and fillets (codes 18301001–18301087)—and sausages made from pork, chicken or beef (codes 18501001–18503009). ‘Easily Swappable Dairy Milk’ was defined as fluid cows’ milk (codes 19101001–19105005). The mean intake of ‘Easily Swappable Animal-Source Meat and Dairy Milk’ for the Australian population aged 2 years and over and other sub-populations of interest were calculated and reported in Table 1. ‘Key Nutrients’ were defined as nutrients for which ‘Easily Swappable Dairy Milk and Animal-Source Meat’ supplied at least 5% of the mean total dietary intake for the Australian population aged 2 years and over (Table 2).

### 2.3. Development of the Dietary Transition Scenarios

For each food category (‘Easily Swappable Animal-Source Meat’ and ‘Easily Swappable Dairy Milk’), two scenarios with differing levels of dietary transition were developed: (1) Accelerated; and (2) Conservative. The Accelerated and Conservative dietary transition scenarios for meat were based on the 2030 projected mean annual intake estimates for plant-based ‘meat’ from two Deloitte Access Economics forecasts [33]. Mean plant-based ‘meat’ consumption was up to 15.5 kg per person/year (≈42 g per person per day) in the Accelerated Meat Scenario and 2.4 kg per person/year (≈6.5 g per person per day) in the Conservative Meat Scenario. To achieve this, in our dietary modelling, ‘Easily Swappable Animal-Source’ beef and chicken were replaced with ‘meat alternative, protein (soy/wheat/pea) base’, a recent addition to Australia’s nutrient survey database [34] and ‘Easily Swappable Animal-Source’ sausages were replaced with plant-based ‘sausages’ (Supplementary Materials; Table S1). The relative proportions of beef, chicken and sausages (39.5, 38.1 and 22.4, respectively) replaced by plant-based ‘meat’ were derived by analysing the ratio of plant-based ‘chicken,’ ‘beef’ and ‘sausage’ products available in major Australian supermarkets in December 2020 [35]. 

The Accelerated Milk Scenario was the total replacement of ‘Easily Swappable Dairy Milk’ with plant-based ‘milk’ (plain ‘milk alternatives’), which increased mean plant-based ‘milk’ consumption by ≈1 L per person/week (with a corresponding decrease in dairy milk). The Conservative Milk Scenario was based on the 86.7% increase in sales of plant-based ‘milk’ between 2012 and 2020 [5] and equated to an additional 6 g per person per day of plant-based ‘milk’ (with a corresponding reduction in dairy milk). The proportions of soy (47.6%), almond (44.2%) and ‘other’ (8.2%) plant-based ‘milk’ used to replace the ‘Easily Swappable Dairy Milk’ in the dietary modelling were based on market information [5]. Within these types of plant-based ‘milk’ (soy, almond, other), the relative proportions of sub-types (e.g., soy fortified with calcium versus unfortified soy ‘milk’) were similar to those in the Base Case for the Australian population aged 2 years and over. The food database codes for the foods altered in the scenarios are listed in Supplementary Materials Table S1.

### 2.4. Dietary Modelling

The impact of gram-for-gram replacement scenarios of the ‘Easily Swappable Animal-Source Meat’ with plant-based ‘meat’ and of ‘Easily Swappable Dairy Milk’ with plant-based ‘milk’ were modelled using Microsoft Excel (Microsoft Corporation, Redmond, WA, USA). 

In each dietary transition scenario, the relevant foods were replaced and then the mean percentage difference in intake of all ‘Key Nutrients’ between the Base Case (2011–2012 intake) and the modelled scenario were calculated. Undesirable hypothetical changes in nutrient intake over 5% are highlighted in the results tables. 

To contextualize the relative importance of the theoretical changes in the intake of ‘Key Nutrients’ determined in our dietary modelling, our results were considered in relation to previously published estimations of nutrient intake inadequacy. In most cases, this figure was based on the proportion of the Australian population with a Usual Nutrient Intake below the Estimated Average Requirement (EAR) [36]. For nutrients where no EAR has been set, the Suggested Dietary Target for adults was used [37,38]. Calculated Usual Nutrient Intakes were derived from two days of dietary intake data. 

## 3. Results

### 3.1. Combined Meat and Milk Scenarios

**Figure 1:**
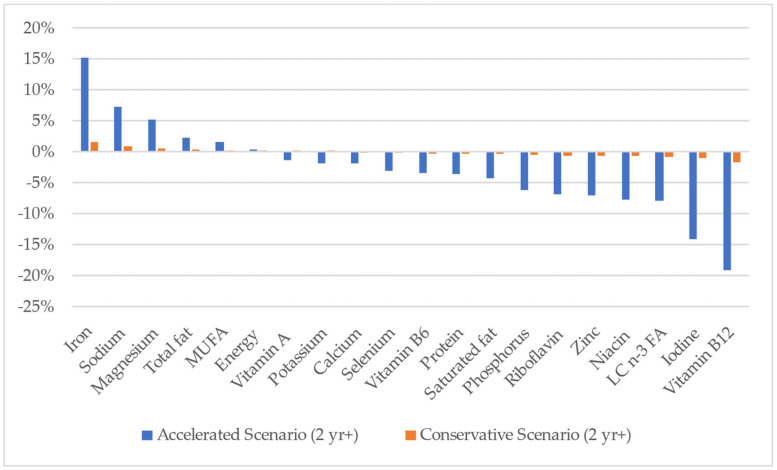
Estimated percentage change ^1^ in mean daily intake of ‘Key Nutrients’ for modelled Combined Meat and Milk Scenarios for the Australian population (2 years and over). ^1^ Compared with Base Case; MUFA, monounsaturated fat; LC n-3 FA, long-chain omega-3 fatty acids.

Figure 1 shows the theoretical changes in the mean intake of the ‘Key Nutrients’ for the Australian population (aged 2 years and over) with the Combined Meat and Milk Scenarios. Theoretical changes in mean intake were less than 2% for all ‘Key Nutrients’ in the Conservative Meat and Milk Scenario but ranged from a 15% increase to a 19% decline in the Accelerated Meat and Milk Scenario. In this scenario, where ≈42 g per person per day of ‘Easily Swappable Animal-source Meat’ was replaced with plant-based ‘meat’ and all ‘Easily Swappable Dairy Milk’ was substituted for plant-based ‘milk’, the most pronounced theoretical decline in mean nutrient intake was for iodine (14%) and vitamin B12 (19%). There was also a theoretical increase in the mean intake of iron, sodium and magnesium of 15%, 7% and 5%, respectively, and a decline in the mean intake of phosphorus, riboflavin (vitamin B2) zinc, niacin and n-3 long-chain fatty acids of 6–8%. 

The predicted impact on the mean nutrient intake of substituting both plant-based ‘meat’ for animal-source meat and plant-based ‘milk’ for dairy milk (combined Accelerated Meat and Milk Scenario) for various population groups are shown in Table 3. In the population groups considered (young children, young women, young men and older adults), there was a theoretical decline in the intake of n-3 long-chain fatty acids (of 7–14%), riboflavin (6–10%), niacin (5–13%), vitamin B12 (16–31%), iodine (12–36%), phosphorus (5–14%) and zinc (5–11%) and an increase in the intake of iron (13–21%), magnesium (5–10%) and sodium (6–10%). In addition, under the Accelerated Milk and Meat Scenario, for young children (but not the other population groups considered), there was a substantial theoretical decline in the intake of protein (8%), vitamin A (7%), calcium (5%), selenium (7%) and potassium (5%). In contrast, the theoretical decline in nutrient intake under the Conservative Milk and Meat Scenario for the population groups considered was small (less than 5%) (Supplementary Materials; Table S2).

Table 4 shows previously reported levels of nutrient intake inadequacy (based on two days of dietary intake) [36,37] alongside results from the Accelerated Meat and Milk Scenario. For iodine, a nutrient that at least 10% of young women were previously estimated to have an inadequate intake of [36], the Accelerated Meat and Milk Scenario modelling predicted a decline in intake of at least 10%. For iodine and vitamin B12 intake by females overall (aged 2 years and over)—a population where 2–10% were previously estimated to have an inadequate usual intake of these nutrients [36]—the Accelerated Meat and Milk Scenario led to a predicted substantial (over 10%) decline in consumption for all subpopulations. For zinc, with the combined scenario, there was a 6% and 8% predicted decline in intake by young men and older adults, respectively, in population groups where 37% of young men and 66% of older men were previously estimated to have an inadequate usual intake [36]. For n-3 long-chain fatty acids for young adults and older adults, riboflavin for older adults, vitamin A for young men, vitamin B6 for young women, calcium and selenium for older adults and protein for older men, over 10% of individuals were previously estimated to be consuming an inadequate intake of these nutrients [36,37], and their intake would theoretically decrease by 2–10% in the Accelerated Meat and Milk Scenario. In contrast, the predicted increased intake of iron and magnesium with the Meat and Milk Scenario occurred in population groups previously reported to have high (over 20%) existing levels of inadequacy [36].

### 3.2. Meat Scenarios

The modelled nutritional impacts of substituting plant-based ‘meat’ for animal-source meat (Accelerated Meat Scenario) with no change in dairy milk intake are listed in Table 5 for the whole population (aged 2 years +) and separate sub-population groups. For the whole population, on average, this switch to the consumption of more plant-based ‘meat’ and less animal-source meat could theoretically result in the increased intake of iron and sodium (of 12% and 6%, respectively) and the decreased intake of n-3 long-chain fatty acids, niacin and vitamin B12 (of 6–7%). Of the sub-population groups considered, the most pronounced theoretical decline in intake of ‘Key Nutrients’ was of n-3 long-chain fatty acids (10%) and niacin (8%) for young women, and of vitamin B12 (8%) for older adults.

The predicted impacts of the Conservative Meat Scenario for various population groups are shown in Supplementary Materials (Table S2)—all changes were less than 5%.

### 3.3. Milk Scenarios

Table 6 shows the theoretical impact on mean nutrient intakes of substituting plant-based ‘milk’ for dairy milk (Accelerated Milk Scenario) with no changes in intake of animal-source meat for the whole population (aged 2 years +) and subgroups of young children, young women, young men and older adults. For the whole population, there was a 3% theoretical increase in iron intake and a decline in the intake of iodine (17%), vitamin B12 (12%), riboflavin, calcium and phosphorus (all ≈5%). Young children would likely experience the most pronounced changes in the intake of ‘Key Nutrients’—an increase of at least 7% for iron and magnesium, a decrease of 5–10% for protein, n-3 long-chain fatty acids, vitamins A, riboflavin, niacin, calcium, potassium and zinc, and a decrease of 13, 24 and 39% for phosphorus, vitamin B12 and iodine, respectively. For the Accelerated Milk Scenario, young men, young women and older adults also had a substantial (10–20%) mean decrease in vitamin B12 and iodine intake.

Supplementary Materials Table S3 shows the predicted impacts of the Conservative Milk Scenario for various population groups—all changes were less than 5%.

## 4. Discussion

Our dietary modelling suggests that the widespread consumption of plant-based ‘meat’ and plant-based ‘milk’ in place of the animal-source originals may negatively impact the intake of some nutrients for the Australian population. In particular, ingestion of nutrients at risk of inadequate intake, such as iodine, vitamin B12, n-3 long-chain fatty acids and zinc, will likely be adversely impacted. Although there were only slight changes in nutrient intake predicted with the Conservative Scenarios (as minor dietary changes were modelled), when the Accelerated Meat and Milk Scenarios were combined, theoretical iron and sodium intake markedly increased (by 15% and 7%, respectively), vitamin B12 and iodine intake declined by 19% and 14%, respectively, and the intake of n-3 long-chain fatty acids, riboflavin, niacin, phosphorus and zinc declined by 6–8%. When modelling the scenarios separately for different population groups and considering our results in conjunction with the previously reported prevalence of nutrient inadequacies from the 2011–12 NNPAS [36], the intake of several nutrients may be of concern due to the modelled reductions in intake potentially exacerbating existing nutritional inadequacies. Examples of these nutrients of concern include the intake of iodine for females, particularly young women, vitamin B12 for females, n-3 long-chain fatty acids for young and older adults, riboflavin for older adults, zinc and vitamin A for young men, vitamin B6 for young women, calcium and selenium for older adults and zinc and protein for older men. The Accelerated Meat Scenario resulted in a 6–7% theoretical reduced intake of n-3 long-chain fatty acids, vitamin B12 and niacin, and an intake of iron and sodium that was 12% and 6% higher, respectively, on a whole population basis. In contrast, the Accelerated Milk Scenario led to a substantial theoretical reduction in the intake of iodine and vitamin B12 (of 17% and 12%, respectively) and a 5% lower intake of riboflavin, calcium and phosphorus.

Previously published studies from the Netherlands which modelled population-wide replacement of animal-source meat and dairy foods with a more varied selection of plant-based alternatives than those used in our study have also reported hypothetical increased iron intake and reduced intake of zinc, vitamin B12 and vitamin A for adults 19–69 years [39] and children aged 2–6 years [26]. Similarly, when modelling the replacement of animal-source meat and dairy milk with plant-based imitation products within an omnivore reference diet for a 20–49-year-old man in the United States, Tso and Forde [28] reported increased sodium intake and decreased intake of vitamin B12, calcium, potassium, magnesium and zinc. However, changes in iodine and n-3 long-chain fatty acids intake were not evaluated in these studies [26,28,39].

The present results reveal that the nutritional implications of swapping animal-source meat and dairy milk for plant-based imitation products are not identical for all population groups and support the need for the consideration of individual variability when making dietary recommendations [40]. Further surveillance of the dietary adequacy of young children, older adults and women of childbearing age may be particularly necessary due to the existing levels of nutritional inadequacy in older adults and women of childbearing age and the reliance on dairy milk as a source of nutrients in young children [40]. As such, the results of this study highlight the need to consider the implications for various population groups, particularly the young, the elderly and women of childbearing age when developing nutrition policies and messages related to transitioning to more environmentally sustainable diets.

One of the key findings of the present modelling study was the substantial (≈14%) predicted decline in Australian iodine intake, in a population already classified as being mildly deficient according to World Health Organization criteria [41]. Iodine is an essential component of thyroid hormones which is needed for normal growth and development, and it is critical for foetal and early life brain development [38]. Despite recent public health measures, Australian women continue to be at risk of iodine deficiency during pregnancy [42,43]. Concerns about the use of unfortified plant-based ‘milks’ leading to an increased risk of iodine deficiency have been raised in the UK [44] and in other high-income countries [45]. Although the UK has seen a small increase in the proportion of plant-based ‘milks’ fortified with iodine between 2015 and 2020 (6% vs. 20%) [44], a 2019–2020 Australian supermarket survey found that less than 1% of plant-based ‘milks’ sold in Australia were fortified with iodine [15], possibly due to the 2009 national recall of a brand of kombu (seaweed)-containing soy ‘milk’ after it caused several cases of iodine toxicity [46].

Animal-source meat and dairy milk are important sources of vitamin B12, and the consumption of dairy milk is associated with a reduced risk of low vitamin B12 status [47,48]. Therefore, the predicted magnitude of the decline in vitamin B12 intake associated with the Accelerated Meat and Milk Scenarios (≈16–19% in adults, ≈31% in young children) compared with 2011–12 mean intakes is concerning. This is particularly the case for older adults, due to their reduced ability to absorb vitamin B12, and for females aged 14 years and over (as 5–8% were previously reported to have an inadequate usual intake in 2011–12) [36]. Our findings support Lamers’ call for a re-evaluation of vitamin B12 requirements and practice guidelines during the transition to more plant-based diets [49].

With an estimated 80% of Australian adults already consuming less than the recommended intake of n-3 long-chain fatty acids in 2011–12 [37], the predicted 6–10% reduction in intake under the Accelerated Meat Scenario modelling is likely to have an adverse impact on various aspects of health [50]. The decline is largely due to meat being an important source of n-3 long-chain fatty acids for the Australian population, particularly for those consuming little or no fish [51,52]. Kesse-Guyot and colleagues [53] have also identified n-3 long-chain fatty acid requirements as being potentially challenging to meet using plant-based diets, although smaller changes in n-3 long-chain fatty acids would likely occur in countries where meat has a lower n-3 long-chain fatty acid content due to the animals consuming more grains and less grass [54,55].

Our results suggest that zinc intake may be a particular concern for males if their dietary intake of animal-source food is reduced, as one in three Australian males and two in three older men have previously been reported to be consuming an inadequate amount in 2011–2012 [36], and the Accelerated Meat and Milk Scenario predicted a 6–8% reduction in its intake. This is consistent with modelling by Dussiot and colleagues showing the inclusion of sufficient bioavailable zinc to be a key challenge in the development of more plant-based diets [56]. With factors in animal-source meat enhancing zinc absorption [57] and phytates in plant ‘meat’ reducing zinc absorption [58], maintenance of an adequate zinc status requires consideration during the development of policies promoting the dietary transition to more plant-based diets, particularly for men.

The increase in iron intake in the Accelerated Scenarios was due to a higher level of fortification of the plant-based meat with non-haem iron than the naturally occurring levels of haem iron in animal-source meat. However, as non-haem iron is less bioavailable than haem iron, and as there are high levels of phytates present in plant ‘meat’ based on soy, pea and wheat protein [58], the increased iron intake may not necessarily improve the population’s iron status. Similarly, the adequacy of the calcium provided by plant ‘milks’ depends on the level of fortification, the additive used and its calcium bioavailability, and the presence of anti-nutritional factors such as phytates [59,60]. According to a 2019–2020 Australian survey of plant-based ‘milks’, both the levels of fortification and the salts used vary widely from product to product [15]. In summary, it should not be assumed that fortifying plant-based ‘meat’ and ‘milk’ with equivalent amounts of iron and calcium to that naturally present in animal-source meat and dairy milk will result in nutritional equivalence for these nutrients.

Surveys indicate that although consumers perceive plant-based ‘milk’ and ‘meat’ to be healthy, they are unaware that these products are nutritionally different to dairy milk and animal-source meat [61,62]. Even if consumers are aware of this, achieving nutritional equivalence is complex, as fortification practices vary between products, and animal-source meat and dairy milk are a key source of a wide range of nutrients—not just the nutrients listed on food labels, such as protein, iron and calcium. In light of the present results, and recent increases in the intake of plant-based ‘milk, it may be timely to re-consider if calcium fortification alone is an adequate criterion for the inclusion of plant ‘milks’ in the dairy group of Australia’s food selection guide [40] and which other nutrients should also be considered. With the current widespread plant-based meat availability and intake likely to increase, the development of recommendations related to its consumption for the population overall and for specific population groups would be a worthwhile inclusion in future dietary guidelines.

Strengths of the present study include the use of a national nutritional composition database (including the 2019 nutritional composition data for ‘meat alternatives: soy/wheat/pea base’ [34]) rather than food labels for dietary modelling, as food labels are often less accurate [63,64]. The national food composition database also enabled a larger range of nutrients to be considered beyond those listed on food labels. The reduction in meat and dairy modelled in this study is a realistic possibility in line with suggested targets and sales data [65,66,67]. In terms of limitations, we did not consider plant-based ‘cheese’ or ‘yoghurt’ in the dietary scenarios, and some consumers may switch all their dairy products to plant-based versions, not just milk. This may have led to us slightly underestimating future changes in the intake of some nutrients. However, in 2020–2021, the mean apparent consumption of plant ‘cheese’ and plant ‘yoghurt’ were substantially lower than that for plant ‘milk’ in Australia (0.0 and 0.3 vs. 17.3 g daily per capita, respectively) [9]. Second, although we attempted to model realistic scenarios based on economic forecasts, surveys, market statistics and recommended dietary transitions to mitigate climate change, the magnitude and speed of dietary change is difficult to predict, and it is unlikely to be uniform among different population groups. For example, a 2022 survey found that young adults aged 18–34 years were more likely to consume plant-based ‘meat’ than older adults [68]. Third, as most types of almond and rice ‘milk’ are required to carry an advisory statement recommending that the product is ‘not suitable as a complete milk replacement for children under 5 years old’ [69], the ratio of soy: almond: other plant ‘milks’ modelled for milk replacement, based on market statistics, may be slightly different for 2- to 3-year-olds than for other population groups, although precise figures are unavailable.

With young adults—the next generation of parents—currently more likely to consume plant-based ‘meat’ and plant-based ‘milk’ than older adults [68,70], further studies are needed to research and monitor the nutritional adequacy of their and their children’s diets in the longer term. Our findings highlight the importance of promoting a wide variety of food sources to meet nutrient needs when consuming a plant-based diet, rather than simply replacing animal source meat and milk products with plant-based alternatives that are similar in appearance. Further studies specifically focusing on pregnant and breast-feeding women, young children and elderly adults and examining the impact of switching from animal-source meat and dairy milk to plant-based ‘milk’ and ‘meat’ are also needed to help identify interventions aimed at ensuring nutritional adequacy. In Australia, it may be worth considering the extension of iodine intake monitoring beyond compliance with mandatory bread fortification to also include changes due to consumers switching from dairy milk to plant ‘milk’. Ideally, future research studies assessing the transition to more sustainable diets should consider a wide range of nutrients, not just those listed on food labels.

## 5. Conclusions

In conclusion, this dietary modelling study assessing the theoretical nutritional implications for the Australian population of substituting plant-based foods that visually and functionally mimic animal-source meat and dairy milk in place of the animal-source ‘originals’ (in combination and separately) suggests that switching to plant-based ‘milk’ and plant-based ‘meat’ may adversely impact the intake of some nutrients. Although sometimes described being beneficial for both health and the environment, the widespread replacement of dairy milk and animal-source meat with plant-based versions may increase the risk of some nutritional inadequacies in the Australian population.

## Figures and Tables

**Table 1:** Base case mean consumption of ‘Easily Swappable Animal-Source Meat’ and ‘Easily Swappable Dairy Milk’ in the Australian National Nutrition and Physical Activity Survey 2011–12.

	Food and AUSNUT Codes [33]	Base Case Mean Daily Intake (g)				
		Total Population (2 Years and Over)	Young Children (2–3 Years)	Young Women (19–30 Years)	Young Men (19–30 Years)	Older Adults (71 Years and Over)
‘Easily Swappable Animal-Source Meat’	Beef 18101001–18101273 (mainly steak, fillet and mince)	18.7	6.9	14.2	22.2	19.4
	Chicken 18301001–18301087 (mainly breast, drumsticks, wings, thighs and fillets)	24.3	12.7	20.9	42.5	21.4
	Sausages 18501001–18503009 (beef, chicken or pork sausages)	10.2	8.0	6.8	13.4	9.3
	Total meat (beef, chicken and sausages)	53.2	27.5	41.9	78.1	50.2
‘Easily Swappable Dairy Milk’	Dairy milk 19101001–19105005 (fluid cows’ milk)	143.4	274.2	115.0	158.7	147.9

Sources: [31,32].

**Table 2:** ‘Key Nutrients’ ^1^ supplied by ‘Easily Swappable Dairy Milk and Animal-Source Meat’ in the Australian National Nutrition and Physical Activity Survey 2011–2012 (for persons aged 2 years and over).

Nutrient	‘Easily Swappable Animal-Source Meat’ ^2^		‘Easily Swappable Dairy Milk’ ^3^		Combined ‘Easily Swappable Dairy Milk and Animal-Source Meat’	
	Mean Intake	% Total Intake	Mean Intake	% Total Intake	Mean Intake	% Total Intake
Energy (kJ)	441	5.2	365	4.3	806	9.5
Protein (g)	13.4	15.3	5.3	6.1	18.7	21.4
Fat (g)	5.5	7.5	3.6	5.0	9.1	12.5
Saturated fat (g)	2.0	7.2	2.4	8.4	4.4	15.6
Monounsaturated fat (g)	2.5	9.0	0.9	3.4	3.4	12.4
n-3 long-chain fatty acids (mg)	21.4	8.6	2.3	0.9	23.7	9.5
Vitamin A retinol equivalents (µg)	7.7	1.0	51.9	6.4	59.6	7.4
Riboflavin (mg)	0.1	4.8	0.3	15.8	0.4	20.6
Niacin derived equivalents (mg)	5.3	13.5	1.5	3.8	6.8	17.3
Vitamin B_6_ (mg)	0.2	10.7	0.1	5.7	0.2	16.4
Vitamin B_12_ (µg)	0.6	13.9	0.9	20.7	1.5	34.6
Calcium (mg)	5.6	0.7	170.0	21.1	175.6	21.8
Iodine (µg)	1.0	0.6	33.1	19.3	34.1	19.9
Iron (mg)	0.8	7.1	0.0	0.3	0.8	7.4
Magnesium (mg)	13.0	4.1	16.4	5.1	29.3	9.2
Phosphorus (mg)	125	8.8	142	10.0	267	18.8
Potassium (mg)	160	5.7	228	8.1	388	13.9
Selenium (µg)	9.7	11.3	1.6	1.9	11.3	13.2
Sodium (mg)	131	5.5	58.9	2.4	190	7.9
Zinc (mg)	1.6	15.5	0.5	5.2	2.2	20.7

^1^ Nutrients for which at least 5% of total intake was supplied by ‘Easily Swappable Dairy Milk and Animal-Source Meat’; ^2^ beef steak, fillet and mince (codes 18101001–18101273), various cuts of chicken—mainly breast, drumsticks, wings, thighs and fillets (codes 18301001–18301087)—and sausages made from pork, chicken or beef (codes 18501001–18503009); ^3^ fluid cows’ milk (codes 19101001–19105005).

**Table 3:** Estimated percentage change ^1^ in mean daily intake of key nutrients if ≈42 g per person per day of ‘Easily Swappable Animal-Source Meat’ and all ‘Easily Swapable’ Dairy Milk is replaced with plant-based ‘meat’ and ‘milk’ (Accelerated Combined Meat and Milk Scenario).

	Population Group				
	All (2 yr+)	Young Children (2–3 yrs)	Young Men (19–30 yrs)	Young Women (19–30 yrs)	Older Adults (71+ yrs)
Energy	0.4	−2.3	0.1	0.7	0.7
Protein	−3.5	−8.1	−2.6	−3.6	−4.5
Total fat	2.3	−1.5	1.6	2.9	3.7
Saturated fat	−4.3	−19.5	−4.6	−2.6	−3.8
Monounsaturated fat	1.6	1.2	1.1	1.8	2.7
n-3 long-chain fatty acids	−7.9	−13.5	−6.5	−10.5	−7.1
Vitamin A (ret. equiv)	−1.4	−7.0	−2.9	−0.8	0.0
Riboflavin	−6.8	−9.8	−6.8	−5.5	−6.5
Niacin (der. equiv)	−7.7	−12.7	−5.4	−9.7	−8.9
Vitamin B6	−3.5	6.7	−3.7	−5.1	−1.0
Vitamin B12	−19.0	−31.3	−16.4	−16.5	−19.7
Calcium	−1.9	−5.0	−1.9	−0.6	−2.4
Iodine	−14.1	−36.4	−13.0	−11.9	−15.4
Iron	15.2	20.9	12.6	16.7	16.2
Magnesium	5.3	10.3	4.9	5.2	5.5
Phosphorus	−6.2	−14.1	−5.2	−6.0	−7.2
Potassium	−1.8	−5.1	−1.6	−1.9	−2.4
Selenium	−3.1	−6.8	−2.3	−4.4	−3.6
Sodium	7.3	9.5	6.1	8.1	9.0
Zinc	−7.0	−11.0	−6.1	−5.1	−8.3

^1^ Compared with Base Case; Shading: undesirable changes of 5–10% are shown in amber; >10–20%, in pink; over 20%, in red.

**Table 4:** Combined Accelerated Meat and Milk Scenario results for various population groups in relation to levels of nutrient intake inadequacy reported in the 2011–12 Australian National Nutrition and Physical Activity Survey.

Predicted Change with the Combined Accelerated Meat and Milk Scenario	Previously Published Estimations of Nutrient Intake Inadequacy in the Australian Population **			
	Less Than 2%	2 to <10%	10 to 20%	Over 20%
Over 20% increase in intake			Iron (YC)	
10 to 20% increase in intake	Magnesium (YC)	Iron (YM, OA)	Iron (All)	Iron (YW)
2 to <10% increase in intake	Vitamin B6 (YC)			Magnesium (All, YA, OA)
Less than 2% change in intake			Vitamin A (All, YW, OA)	Vitamin B6 (OA) Calcium (All, YM, YW)
2 to <10% decrease in intake	Riboflavin (YC) Niacin (All, YM, YW, OA) Phosphorus (All M, YM, YW, OA) Protein (All, 2–3, YM, YW) Vitamin A (YC) Calcium (YC) Selenium (YC, YM)	Riboflavin (All M, All F, YM, YW) Phosphorus (All F) Protein (OW) Vitamin B6 (All, YM) Selenium (All, YW)	Protein (OM) Zinc (All F, YW, OW) Selenium (OA)	n-3 long-chain fatty acids (YA, OA) Riboflavin (OA) Zinc (All M, YM, OM) Vitamin A (YM) Vitamin B6 (YW) Calcium (OA)
10–20% decrease in intake	Niacin (YC) Vitamin B12 (All M, YM, OM) Iodine (All M, YM) Phosphorus (YC) Zinc (YC)	Vitamin B12 (All F, YW, OW) Iodine (All F, OA)	Iodine (YW)	
Over 20% decrease in intake	Vitamin B12 (YC) Iodine (YC)			

** Proportion of the Australian population with a Usual Nutrient Intake below the Estimated Average Requirement (EAR) [36] (or where no EAR has been set, the Suggested Dietary Target (SDT)); All = population aged 2 years and over, All M = all males aged 2 years and over, All F = all females aged 2 years and over, YC = children aged 2–3 years, YM = young men (19–30 y), YW = young women (19–30 y), YA = young adults (19–30 y), OA = older adults (71 y and over), OM = older men (71 y and over), OW = older women (71 y and over), (male/female differences shown where inadequacies vary between males and females). Shading: desirable changes show in green, undesirable changes in light pink to red, the redder the shade, the more undesirable.

**Table 5:** Estimated percentage change ^1^ in mean daily intake of ‘Key Nutrients’ if ≈42 g per person per day of ‘Easily Swappable Animal-Source Meat’ is replaced with plant-based ‘meat’ (Accelerated Meat Scenario).

	Population Group				
	All (2 Years+)	Young Children (2–3 Years)	Young Men (19–30 Years)	Young Women (19–30 Years)	Older Adults (71+ Years)
Energy	1.2	1.0	0.9	1.4	1.4
Protein	−1.0	−0.7	−0.7	−1.3	−1.2
Total fat	2.4	1.8	1.9	2.8	3.0
Saturated fat	2.2	1.3	1.7	2.9	2.6
Monounsaturated fat	0.3	−0.3	0.2	0.5	0.4
n-3 long-chain fatty acids	−7.1	−8.5	−5.7	−9.5	−6.5
Vitamin A (ret. equiv)	0.3	0.1	0.3	0.3	0.2
Riboflavin	−1.7	−1.2	−1.1	−1.9	−1.9
Niacin (der. equiv)	−5.7	−7.1	−4.0	−7.9	−6.3
Vitamin B6	−3.6	−4.7	−2.3	−4.8	−4.4
Vitamin B12	−7.4	−6.9	−5.6	−6.5	−8.1
Calcium	3.5	2.4	3.0	3.6	4.1
Iodine	3.3	2.5	2.7	3.7	3.6
Iron	11.8	11.6	9.5	13.7	12.6
Magnesium	3.2	3.3	2.6	3.3	3.6
Phosphorus	−0.9	−0.8	−0.8	−1.1	−0.9
Potassium	0.6	0.8	0.5	0.5	0.7
Selenium	−2.4	−3.6	−1.8	−3.8	−2.8
Sodium	6.4	6.3	5.3	7.3	7.9
Zinc	−3.9	−2.5	−3.6	−2.2	−4.6

^1^ Compared with Base Case; Shading: undesirable changes of 5–10% are shown in amber.

**Table 6:** Estimated percentage change ^1^ in mean daily intake of ‘Key Nutrients’ if all ‘Easily Swappable Dairy Milk’ is replaced with plant-based ‘milk’ (Accelerated Milk Scenario).

	Population Group				
	All (2 Years+)	Young Children (2–3 Years)	Young Men (19–30 Years)	Young Women (19–30 Years)	Older Adults (71+ Years)
Energy	−0.8	−3.3	−0.8	−0.7	−0.7
Protein	−2.6	−7.4	−1.9	−2.3	−3.2
Total fat	−0.1	−3.3	−0.2	0.0	0.7
Saturated fat	−6.5	−20.7	−6.3	−5.5	−6.4
Monounsaturated fat	1.3	1.5	0.9	1.2	2.3
n-3 long-chain fatty acids	−0.8	−5.0	−0.8	−0.9	−0.6
Vitamin A (ret. equiv)	−1.7	−7.2	−3.2	−1.1	−0.2
Riboflavin	−5.1	−8.7	−5.7	−3.6	−4.7
Niacin (der. equiv)	−2.0	−5.6	−1.4	−1.8	−2.5
Vitamin B6	0.1	11.4	−1.4	−0.3	3.4
Vitamin B12	−11.7	−24.3	−10.8	−10.0	−11.6
Calcium	−5.4	−7.4	−4.9	−4.2	−6.5
Iodine	−17.4	−38.9	−15.7	−15.6	−19.1
Iron	3.4	9.2	3.2	2.9	3.6
Magnesium	2.1	7.0	2.3	1.9	2.0
Phosphorus	−5.2	−13.2	−4.4	−4.8	−6.3
Potassium	−2.4	−5.9	−2.2	−2.4	−3.1
Selenium	−0.7	−3.2	−0.5	−0.7	−0.8
Sodium	0.9	3.1	0.8	0.7	1.1
Zinc	−3.1	−8.5	−2.4	−2.9	−3.7

^1^ Compared with Base Case; Shading: undesirable changes of 5–10% are shown in amber, >10–20%, in pink, over 20%, in red.

## Data Availability

The data modelled are from the following publicly available sources: the Australian Bureau of Statistics [35] and Food Standards Australia New Zealand [33].

## Supplementary Materials

The following supporting information can be downloaded at: https://www.mdpi.com/article/10.3390/nu15081825/s1, Table S1: Food codes for plant-based ‘meat’ and plant-based ‘milk’; Table S2: Estimated percentage change ^1^ in mean daily intake of key nutrients if ≈6.5 g per person per day of ‘Easily Swappable Animal-Source Meat’ and 6 g per person per day ‘Easily Swapable’ Dairy Milk is replaced with plant-based ‘meat’ and ‘milk’ (Conservative Combined Meat and Milk Scenario); Table S3: Estimated percentage change ^1^ in mean daily intake of ‘Key Nutrients’ if ≈6.5 g per person per day of ‘Easily Swappable Animal-Source Meat’ is replaced with plant-based ‘meat’ (Conservative Meat Scenario); Table S4: Estimated percentage change ^1^ in mean daily intake of ‘Key Nutrients’ if ≈6 g per person per day of ‘Easily Swappable Dairy Milk’ is replaced with plant-based ‘milk’ (Conservative Milk Scenario).
