# Supplementary material for: Impact of a Switch to Plant-Based Foods That Visually and Functionally Mimic Animal-Source Meat and Dairy Milk for the Australian Population—A Dietary Modelling Study
Source: Nutrients. 2023 Apr 10;15(8):1825. doi: 10.3390/nu15081825 (PMC10147004; doi:10.3390/nu15081825)
Supplement: Supplementary file 1 [file nutrients-15-01825-s001.zip › nutrients-2270379-supplementary.pdf]

Supplementary Material

Table S1: Food codes [1] for the plant-based 'meat' and plant-based 'milk' used in the dietary modelling

Food	**Code**
Plant-based meat	F009823
Plant-based sausage	20601004
Soy beverage	20101002
	20101003
	20102001
	20102002
	20102003
	20102004
	20103001
	20104001
	20104002
	20104003
	20104004
	20104005
	20105001
	20107003
	20107004
	20107005
Almond 'milk'	20106001
Other (Oat 'milk' and rice 'milk)	20106002
	20106003
	20106004
	20106005
	20107002

From the Australian Food Composition Database (Release 2) and the Australian Food, Supplement and Nutrition Database (AUSNUT) 2011-13

Table S2: Estimated percentage change[1] in mean daily intake of key nutrients if ≈6.5g per person per day of 'Easily Swapable Animal-Source Meat' and 6g per person per day 'Easily Swapable' Dairy Milk is replaced with plant-based 'meat' and 'milk' (Conservative Combined Meat and Milk Scenario).

	Population group				
	**All (2 years+)**	**Young children (2-3 years)**	**Young men (19-30 years)**	**Young women (19-30 years)**	**Older adults (71+ years)**
Energy	0.1%	0.0%	0.1%	0.0%	0.1%
Protein	-0.3%	-0.7%	-0.2%	-0.5%	-0.4%
Total fat	0.4%	0.4%	0.3%	0.3%	0.5%
Saturated fat	0.0%	-0.4%	0.1%	-0.3%	0.1%
Monounsaturated fat	0.2%	0.3%	0.1%	0.3%	0.3%
n-3 long chain fatty acids	-1.0%	-2.4%	-0.8%	-1.3%	-1.0%
Vitamin A (ret eq)	-0.2%	-0.6%	-0.1%	-0.4%	-0.2%
Riboflavin	-0.7%	-1.1%	-0.4%	-1.1%	-1.0%
Niacin (der eq)	-0.9%	-1.7%	-0.6%	-1.1%	-1.1%
Vitamin B6	-0.7%	-1.3%	-0.4%	-0.8%	-0.9%
Vitamin B12	-1.7%	-3.2%	-1.2%	-2.5%	-2.1%
Calcium	0.2%	0.0%	0.3%	-0.1%	0.1%
Iodine	-0.3%	-0.9%	0.0%	-1.1%	-0.3%
Iron	1.6%	2.4%	1.4%	1.7%	1.9%
Magnesium	0.5%	0.8%	0.4%	0.5%	0.5%
Phosphorus	-0.4%	-0.9%	-0.3%	-0.7%	-0.5%
Potassium	-0.2%	-0.4%	-0.1%	-0.4%	-0.2%
Selenium	-0.4%	-0.7%	-0.3%	-0.4%	-0.4%
Sodium	0.9%	1.7%	0.8%	0.9%	1.2%
Zinc	-0.7%	-1.4%	-0.6%	-0.7%	-0.8%

Table S3: Estimated percentage change[1] in mean daily intake of 'Key Nutrients' if ≈6.5g per person per day of 'Easily Swapable Animal-Source Meat' is replaced with plant-based 'meat' (Conservative Meat Scenario)

	**Population group**				
	**All (2 years+)**	**Young children (2-3 years)**	**Young men (19-30 years)**	**Young women (19-30 years)**	**Older adults (71+ years)**
Energy	0.2%	0.2%	0.1%	0.2%	0.2%
Protein	-0.1%	-0.3%	-0.1%	-0.1%	-0.2%
Total fat	0.3%	0.4%	0.3%	0.3%	0.4%
Saturated fat	0.3%	0.3%	0.2%	0.3%	0.4%
Monounsaturated fat	0.0%	0.0%	0.0%	0.0%	0.1%
n-3 long chain fatty acids	-1.0%	-2.2%	-0.8%	-1.2%	-0.9%
Vitamin A (ret eq)	0.0%	0.1%	0.0%	0.0%	0.0%
Riboflavin	-0.2%	-0.2%	-0.2%	-0.2%	-0.3%
Niacin (der eq)	-0.8%	-1.4%	-0.6%	-0.8%	-0.9%
Vitamin B6	-0.5%	-0.8%	-0.3%	-0.5%	-0.6%
Vitamin B12	-1.0%	-1.7%	-0.8%	-0.9%	-1.2%
Calcium	0.5%	0.5%	0.4%	0.4%	0.6%
Iodine	0.4%	0.5%	0.4%	0.5%	0.5%
Iron	1.6%	2.3%	1.3%	1.6%	1.8%
Magnesium	0.4%	0.7%	0.4%	0.4%	0.5%
Phosphorus	-0.1%	-0.2%	-0.1%	-0.1%	-0.1%
Potassium	0.1%	0.1%	0.1%	0.1%	0.1%
Selenium	-0.3%	-0.6%	-0.3%	-0.4%	-0.4%
Sodium	0.9%	1.6%	0.7%	0.8%	1.1%
Zinc	-0.5%	-1.1%	-0.5%	-0.4%	-0.7%

Table S4: Estimated percentage change[1] in mean daily intake of 'Key Nutrients' if 6g per person per day 'Easily Swapable Dairy Milk' is replaced with plant-based 'milk' (Conservative Milk Scenario)

	Population group				
	**All (2 years+)**	**Young children (2-3 years)**	**Young men (19-30 years)**	**Young Women (19-30 years)**	**Older adults (71+ years)**
Energy	-0.1%	-0.2%	0.0%	-0.1%	-0.1%
Protein	-0.2%	-0.5%	-0.1%	-0.4%	-0.3%
Total fat	0.0%	-0.1%	0.0%	0.0%	0.1%
Saturated fat	-0.3%	-0.8%	-0.2%	-0.5%	-0.3%
Monounsaturated fat	0.1%	0.3%	0.1%	0.3%	0.2%
n-3 long chain fatty acids	0.0%	-0.2%	0.0%	-0.1%	0.0%
Vitamin A (ret eq)	-0.2%	-0.7%	-0.2%	-0.4%	-0.2%
Riboflavin	-0.5%	-0.9%	-0.3%	-0.9%	-0.7%
Niacin (der eq)	-0.1%	-0.3%	-0.1%	-0.2%	-0.2%
Vitamin B6	-0.2%	-0.6%	-0.1%	-0.3%	-0.3%
Vitamin B12	-0.7%	-1.5%	-0.4%	-1.6%	-1.0%
Calcium	-0.3%	-0.5%	-0.2%	-0.6%	-0.4%
Iodine	-0.8%	-1.4%	-0.4%	-1.5%	-0.8%
Iron	0.1%	0.1%	0.0%	0.1%	0.0%
Magnesium	0.1%	0.1%	0.0%	0.1%	0.0%
Phosphorus	-0.3%	-0.7%	-0.2%	-0.6%	-0.4%
Potassium	-0.2%	-0.5%	-0.1%	-0.5%	-0.3%
Selenium	0.0%	-0.1%	0.0%	-0.1%	0.0%
Sodium	0.0%	0.1%	0.0%	0.1%	0.1%
Zinc	-0.1%	-0.3%	-0.1%	-0.3%	-0.2%
